# Die Bedeutung von Sarkopenie für die immunvermittelte Toxizität bei Patienten mit malignem Melanom unter einer Immuncheckpoint-Inhibition

**DOI:** 10.1007/s00105-024-05405-9

**Published:** 2024-08-14

**Authors:** Christian Holtorf, Miriam Mengoni, Thomas Tüting, Andreas Wienke, Jan Borggrefe, Alexey Surov, Mareike Alter

**Affiliations:** 1 Hautarztpraxis Dr. med. Anke Raschke, Magdeburg, Deutschland; 2 Universitätsklinik für Dermatologie und Venerologie, Universitätsklinikum Magdeburg, Magdeburg, Deutschland; 3 Universitätsklinik für Dermatologie und Venerologie, Ruhr-Universität Bochum, Campus Minden, Johannes-Wesling-Klinikum Minden, Hans-Nolte-Str. 1, 32429 Minden, Deutschland; 4 Institut für Medizinische Epidemiologie, Biometrie und Informatik, Martin-Luther-Universität, Halle-Wittenberg, Deutschland; 5 Universitätsinstitut für Radiologie, Neuroradiologie und Nuklearmedizin, Johannes-Wesling Klinikum, Ruhr-Universität Bochum, Minden, Deutschland

**Keywords:** Sarkopenie, Malignes Melanom, Immuncheckpoint-Inhibition, Toxizität, Psoasmuskulatur, Muscular atrophy, Malignant melanoma, Immune checkpoint inhibitors, Toxicity, Psoas muscles

## Abstract

**Hintergrund:**

Sarkopenie ist die Verminderung der Muskelkraft und -masse sowie Einschränkung der Funktion. Das Ziel der vorliegenden Studie war es zu untersuchen, ob die anhand der Psoasmuskulatur bestimmte prätherapeutische Sarkopenie die therapievermittelte Toxizität bei Patienten mit malignem Melanom unter einer Immuncheckpoint-Inhibition beeinflusst.

**Patienten und Methoden:**

Die Vermessung der Psoasmuskulatur erfolgte prätherapeutisch mithilfe der Computertomographie auf der Höhe des 3. Lendenwirbelkörpers (LWK) bei 75 Patienten zwischen Januar 2011 und Dezember 2020. Die Sarkopenie wurde anhand des Psoasmuskelindex (PMI) definiert. Die immunvermittelte Toxizität wurde retrospektiv ermittelt.

**Ergebnisse:**

Bei 33 der 75 Patienten (44 %) wurde eine behandlungsbedingte Toxizität unter Therapie mit Immuncheckpoint-Inhibitoren registriert. Davon erlitten 16 Patienten (36,2 %) eine dosislimitierende schwere Toxizität (DLT). Eine prätherapeutische Sarkopenie wurde bei 25 Patienten (33,3 %) ermittelt. Die Vergleichsanalyse ergab, dass die Patienten mit einer DLT im Vergleich zu der Patientengruppe ohne DLT niedrigere PMI-Werte aufwiesen (4,65 ± 1,33 vs. 5,79 ± 1,67 cm^2^m^−2^, *p* = 0,015) (OR = 0,60, 95 %-KI: 0,40–0,92, *p* = 0,02).

**Schlussfolgerungen:**

Die anhand der Psoasmuskulatur gemessene prätherapeutische Sarkopenie ist kein signifikanter Prädiktor für DLT bei Patienten mit malignem Melanom unter einer Immuncheckpoint-Inhibition. Patienten mit einer DLT weisen jedoch im Vergleich zu der Patientengruppe ohne DLT niedrigere Werte für die Psoasmuskelparameter PMI und Gauge auf.

## Abstract (en)

**Background and objectives:**

Sarcopenia is the generalized loss of muscle strength, mass, and function. The aim was to investigate whether pretherapeutic sarcopenia, as determined by the psoas muscles, affects therapy-mediated toxicity in patients with malignant melanoma undergoing immunotherapy.

**Patients and methods:**

Measurement of psoas musculature was performed pretherapeutically using computed tomography at the level of the third lumbar vertebra in the axial plane in 75 patients between January 2011 and December 2020. Sarcopenia was defined using the psoas muscle index (PMI). Immune-related toxicity was retrospectively assessed.

**Results:**

Treatment-related toxicity was recorded in 33 of the 75 patients (44%). Of these, 16 patients (36.2%) experienced dose-limiting severe events (DLT). Pretherapeutic sarcopenia was identified in 25 patients (33.3%). Comparative analysis showed that the patients with a DLT had lower PMI values compared with the patient group without a DLT (4.65 ± 1.33 vs. 5.79 ± 1.67 cm^2^m^−2^, *p* = 0.015) (odds ratio = 0.60, 95% confidence interval 0.40–0.92, *p* = 0.02).

**Conclusions:**

Pretherapeutic sarcopenia measured based on the psoas muscle is not a significant predictor of immune-mediated toxicity in patients with malignant melanoma treated with immune checkpoint inhibitors. Patients with DLT have lower values for the psoas muscle parameters PMI compared to the group of patients without DLT.

## Hintergrund

Immuncheckpoint-Inhibitoren (ICI) sind Bestandteil der Therapie bei Patienten mit fortgeschrittenem malignem Melanom. Unter der Therapie mit ICI können immunvermittelte Nebenwirkungen (irAEs) auftreten. Die Prävalenz der Sarkopenie, die durch einen Verlust von Muskelkraft, -masse und -funktion definiert wird, beträgt etwa 39 % bei Krebspatienten vor Therapiebeginn [1, 2]. Sarkopenie ist somit ein häufiges Syndrom bei fortgeschrittenen malignen Erkrankungen [3]. Verschiedene Studien haben Sarkopenie als Prädiktor für operative Komplikationen bestätigt [4, 5]. In der Onkologie wurde ein Zusammenhang zwischen prätherapeutischer Sarkopenie und Toxizität unter Chemotherapie beobachtet [2]. Ein vielversprechender Ansatz ist daher die Vorhersage einer Toxizität bei Patienten mit malignem Melanom vor einer Therapie mit ICI anhand der Sarkopenie als möglichem Prädiktor. In einigen Studien wurde bereits ein Zusammenhang zwischen Sarkopenie und immunvermittelter Toxizität bei Patienten mit malignem Melanom beobachtet [6, 7].

Aktuell existiert keine standardisierte Methode zur Bestimmung der Sarkopenie [1]. Mehrere Autoren konnten allerdings zeigen, dass die gesamte Skelettmuskelmasse mit der Muskelmasse des M. psoas korreliert [8–10].

Im Rahmen dieser Studie wurde untersucht, ob es eine Korrelation zwischen der anhand der Psoasmuskulatur bestimmten Sarkopenie bei Patienten mit malignem Melanom und der unter der Therapie mit ICI auftretenden immunvermittelten Toxizität gibt.

## Methodik

### Datenerhebung und Patienten

Klinische Daten von 78 Patienten mit malignem Melanom im Stadium der Lymphknoten- und Fernmetastasierung wurden retrospektiv anhand der archivierten Patientenakten akquiriert. Die Auswahl der Patienten erfolgte mithilfe einer Zufallsstichprobe innerhalb des Patientenkollektivs, bei denen im Zeitraum zwischen Januar 2011 und Dezember 2020 eine Immuntherapie mit Pembrolizumab, Ipilimumab, Nivolumab oder mit der Kombination aus Ipilimumab und Nivolumab am Universitätsklinikum Magdeburg eingeleitet wurde. Es wurden Patienten eingeschlossen, die im Rahmen einer Erstlinientherapie behandelt wurden, bis maximal zur dritten Behandlungslinie. Von 75 der ursprünglich 78 untersuchten Patienten konnten prätherapeutisch geeignete CT-Bilder akquiriert werden, sodass 23 Patienten mit adjuvanter Therapie und 52 Patienten im inoperablen bzw. fernmetastasierten Stadium eingeschlossen wurden. Die Bestimmung des Schweregrades der irAEs erfolgte anhand der aktuell gültigen Common Terminology Criteria for Adverse Events Version 5.0 (CTCAE v5.0) [11]. Anhand der prätherapeutischen Bilder der Computertomographie erfolgte die Berechnung unterschiedlicher Parameter der Psoasmuskulatur. In der vorliegenden Studie wurde der Psoasmuskelindex (PMI) zur Bestimmung von Sarkopenie verwendet.

### Messung der Psoasmuskulatur im CT

**Abb. 1:**
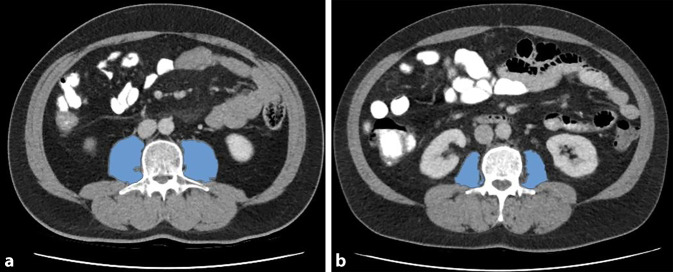
**a** Männlicher Patient ohne Sarkopenie, PMI-Wert: 8,75 cm^2^ m^−2^; **b** männlicher Patient mit Sarkopenie, PMI-Wert: 4,30 cm^2^ m^−2^

Alle computertomographischen (CT) Untersuchungen wurden in der portalvenösen Phase durchgeführt. Die Messungen am linken und rechten M. psoas wurden anhand von CT-Bildern auf der Höhe des 3. LWK in der Axialebene durchgeführt (Abb. 1). Ein Weichteilfenster von −30 bis 110 Hounsfield units (HU) wurde gewählt, um größere Gefäß- und Fettinfiltrationen der Muskeln von den nachfolgenden Berechnungen auszuschließen. Die Psoasmuskelflächen PMA_links_ und PMA_rechts_ wurden addiert, um die gesamte Psoasmuskelfläche (PMA_gesamt_) zu berechnen. Aus dem Mittelwert der berechneten Abschwächung der Röntgenstrahlung (Röntgenopazität) beider Psoasmuskeln wurde die Röntgendichte (PMD) in HU berechnet (Abb. 1).

### Berechnung verschiedener Parameter der Psoasmuskulatur

Ergänzend wurden anhand der bereits bestimmten Parameter der Psoasmuskulatur weitere Parameter errechnet, deren klinische Relevanz bereits in wissenschaftlichen Publikationen unter Beweis gestellt werden konnte: Psoasmuskelindex (PMI) und die Psoas-Gauge [4, 5]. Der PMI wurde berechnet durch Bildung eines Quotienten aus PMA_gesamt_ und der Körpergröße des Patienten. Zudem errechneten wir die Psoas-Gauge, welche den Psoasmuskelindex und die Psoasmuskeldichte kombiniert [12]. Hierfür wurde der PMI mit der PMD multipliziert. Als geschlechtsspezifische Grenzwerte für die Diagnose der Sarkopenie wurden die bereits von anderen Autoren publizierten Werte verwendet: PMI < 5,24 cm^2^/m^2^ für Männer und PMI < 3,85 cm^2^/m^2^ für Frauen [13, 14].

### Immunvermittelte Toxizität unter Immuncheckpoint-Inhibition

Die irAEs wurden nach Durchsicht der digital archivierten Patientenakten dokumentiert und entsprechend der CTCAE v5.0 in die Schweregrade eingeteilt: Grad I–II (leicht bis mittelschwer), III–IV (schwer bis lebensbedrohlich) und V (Tod). Die dosislimitierende Toxizität (DLT) wurde festgelegt als ein irAE mit einem Schweregrad ≥ III (schwer bis tödlich). Sofern bei einem Patienten mehrere irAEs im Nachbeobachtungszeitraum auftraten, wurden diese vollständig erfasst. Hierbei war die schwerste Toxizität ausschlaggebend für die Zuordnung zu einer DLT.

### Statistische Analyse

Die statistische Analyse erfolgte mit dem SPSS-Paket (IBM SPSS Statistics for Windows, v. 225.0, IBM Corporation, Armonk, NY, USA). Die Daten wurden je nach Eignung als Mittelwert ± Standardabweichung oder Median (IQR) angegeben. Der Zusammenhang zwischen der Toxizität und der Sarkopenie wurde anhand einer Vergleichsanalyse, univariaten sowie multivariaten binär-logistischen Regressionsanalyse und der Odds-Ratio ermittelt. Ein *p*-Wert < 0,05 wurde als signifikant betrachtet.

## Ergebnisse

### Eingeschlossene Patienten und Analyse der Muskelparameter

Die Studienpopulation umfasste 43 Männer (57,3 %) und 32 Frauen (42,7 %). Der PMI betrug durchschnittlich 5,54 ± 1,65 cm^2^m^−2^, eine Sarkopenie wurde bei 25 Patienten (33,3 %) ermittelt. Die Charakteristika der Studienpopulation sind in Tab. 1 und die anthropometrischen Maßzahlen und Parameter der Psoasmuskulatur in Tab. 2 dargestellt.

**Tab. 1:** Charakteristika der Studienpopulation, die Werte werden in *n* (%) angegeben

Charakteristika der Studienpopulation	*n* = 75
*Alter (Jahre) bei Erstdiagnose Melanom*	
Median (IQR)	64 (49–75)
Altersspanne	18–87
Alter (Jahre) bei Start der Immuntherapie (IQR)	67 (52–76)
*Geschlecht*	
Männlich	43 (57,3)
Weiblich	32 (42,7)
*Immuncheckpoint-Inhibitor bei Einleitung der Immuntherapie*	
Nivolumab, *n* (%)	30 (40,0)
Pembrolizumab, *n* (%)	26 (34,7)
Ipilimumab, *n* (%)	8 (10,6)
Ipilimumab und Nivolumab, *n* (%)	11 (14,7)
*BRAF-Mutationsstatus zum Zeitpunkt der Immuntherapieeinleitung*	
BRAF-positiv	29 (38,6)
BRAF-negativ	45 (60,0)
BRAF nicht auswertbar	1 (1,3)
*M‑Stadium zum Zeitpunkt der Immuntherapieeinleitung*	
M1a	14 (26,9)
M1b	10 (19,2)
M1c	21 (40,4)
M1d	7 (13,5)
*AJCC-Stadium des malignen Melanoms zum Zeitpunkt der Immuntherapieeinleitung*	
IIIA	2 (2,6)
IIIB	4 (5,3)
IIIC	13 (17,3)
IIID	4 (5,3)
IV	52 (69,3)
*Klinisches Stadium nach AJCC 2017*	
Patienten mit adjuvanter Therapie	23 (30,7)
Patienten mit Therapie im inoperablen Stadium	52 (69,3)

*IQR* Interquartilsabstand, *IrAE* immunvermitteltes unerwünschtes Ereignis, *BRAF* „v-Raf murine sarcoma viral oncogene homolog B1“, *AJCC* American Joint Commitee on Cancer

**Tab. 2:** Anthropometrie und Parameter der Psoasmuskulatur, die Werte werden als Standardabweichung angegeben (SD), sofern nicht anders angegeben

Charakteristik	Männer (*n* = 43)	Frauen (*n* = 32)	Insgesamt (*n* = 75)
BMI (kg m^−2^)	27,25 (3,69)	31,62 (6,27)	27,73 (4,97)
BSA (m^2^)	2,04 (0,17)	1,94 (0,20)	1,96 (0,21)
Untergewicht (≤ 18,5 kg m^−2^), *n* (%)	0 (0)	1 (3,1)	1 (1,3)
Normalgewicht (18,5–24,9 kg m^−2^), *n* (%)	13 (30,2)	9 (28,1)	23 (30,7)
Übergewicht (25–29,9 kg m^−2^) *n* (%)	23 (53,5)	9 (28,1)	32 (42,7)
Adipositas (≥ 30,0 kg m^−2^), *n* (%)	7 (16,3)	13 (40,6)	20 (26,7)
PMA (cm^2^)	19,92 (5,00)	12,41 (3,15)	16,72 (5,69)
PMI (cm^2^ m^−2^)	6,31 (1,54)	4,52 (1,17)	5,54 (1,65)
Sarkopenie, *n* (%)	16 (37,2)	9 (28,1)	25 (33,3)
PMD (HU)	45,78 (7,30)	46,42 (10,04)	46,05 (8,58)
Psoas-Gauge (AU)	307,52 (105,95)	213,95 (85,92)	259,85 (105,04)

*BMI* Body-Mass-Index, *BSA* „body surface area“, *PMA* Psoasmuskelfläche, *PMI* Psoasmuskelindex, *PMD* Psoasmuskeldichte, *HU* Hounsfield unit, *AU* willkürliche Einheit

### Immunvermittelte Toxizität unter einer Therapie mit Immuncheckpoint-Inhibitoren

Bei 33 der 75 Patienten (44 %) wurde eine immunvermittelte Toxizität unter Therapie mit ICI beobachtet. Davon erlitten 17 Patienten (63,8 %) leichte bis mittelschwere unerwünschte Ereignisse und 16 Patienten (36,2 %) dosislimitierende schwere Ereignisse. Insgesamt ereigneten sich bei diesen 33 Patienten kumulativ 50 irAEs. Die Art und der Schweregrad der erfassten irAEs sind in Abb. 1 dargestellt.

### Zusammenhang zwischen Psoasmuskelparametern und immunvermittelter Toxizität

Die Vergleichsanalyse ergab, dass die Patienten mit einer DLT im Vergleich zu der Patientengruppe ohne DLT niedrigere PMI-Werte (4,65 ± 1,33 vs. 5,79 ± 1,67 cm^2^m^2^, *p* = 0,015) sowie eine niedrigere Gauge (210,44 ± 85,43 vs. 273,24 ± 107,35 AU, *p* = 0,034) aufwiesen (OR = 0,60, 95 %-KI: 0,40–0,92, *p* = 0,02; OR = 0,99, 95 %-KI: 0,98–1,00, *p* = 0,04, respektiv) (Tab. 3). In der univariaten Regressionsanalyse zeigte sich, dass Patienten mit einer niedrigeren Gauge eine höhere Inzidenz von DLT hatten, verglichen mit der Patientengruppe mit höherer Gauge (OR = 0,993, 95 %-KI: 0,986–1,000, *p* = 0,04) (Tab. 4). Nach Durchführung einer multivariablen Regressionsanalyse wies allerdings keiner der analysierten Prädiktoren eine statistische Signifikanz hinsichtlich der prognostischen Bedeutung für das Auftreten der DLT auf (Tab. 5).

**Tab. 3:** Vergleichsanalyse zwischen der DLT und Nicht-DLT und den Variablen PMI, PMD, Gauge, BMI und BSA

M ± SD	Keine DLT	DLT	*p*-Wert
PMI	5,79 ± 1,67	4,65 ± 1,33	0,02
PMD	46,45 ± 8,22	44,57 ± 10,19	0,44
Gauge	273,24 ± 107,35	210,44 ± 85,43	0,03
BMI	27,16 ± 6,19	28,06 ± 4,39	0,58
BSA	1,95 ± 0,33	1,89 ± 0,19	0,49
*Vergleichsanalyse der Subgruppen*			
Patienten mit Sarkopenie	18 (30,5 %)	8 (50,0 %)	0,146
Sarkopene Patienten mit Übergewicht	12 (20,0 %)	6 (37,5 %)	0,143

*DLT* dosislimitierende Toxizität, *PMI* Psoasmuskelindex, *PMD* Psoasmuskeldichte, *BMI* Body-Mass-Index, *BSA* Körperoberfläche

**Tab. 4:** Prädiktoren für die DLT. Univariable Regressionsanalyse

		OR	95 %-KI	*p*-Wert
PMI		0,60	0,40–0,92	0,02
PMD		0,97	0,91–1,04	0,44
Gauge		0,99	0,98–1,00	0,04
Sarkopenie vs. keine Sarkopenie		2,28	0,74–7,02	0,15
Sarkopenie mit Übergewicht vs. keine Sarkopenie mit Übergewicht		2,40	0,73–7,92	0,15

*PMI* Psoasmuskelindex, *PMD* Psoasmuskeldichte, *OR* Odds Ratio, *KI* Konfidenzintervall

**Tab. 5:** Prädiktoren für die DLT. Multivariable Regressionsanalyse

		OR	95 %-KI	*p*-Wert
Gauge		0,99	0,98–1,00	0,13
BMI		1,02	0,93–1,13	0,58

*BMI* Body mass index

## Diskussion

Es wird zunehmend anerkannt, dass die Körperzusammensetzung bei vielen Krankheitsbildern einen prognostischen Einflussfaktor darstellt [4, 5]. Diese Studie untersucht, ob die anhand der Psoasmuskulatur gemessene prätherapeutische Sarkopenie die Vorhersage einer immunvermittelten Toxizität bei Patienten mit malignem Melanom unter Therapie mit ICI ermöglicht.

In dieser Studie wurde eine dosislimitierende Toxizität bei 21 % der untersuchten Studienpopulation beobachtet. Die Patientengruppe mit DLT wies im Vergleich zu der Patientengruppe ohne DLT niedrigere Werte für die Psoasmuskelparameter PMI und Gauge auf (OR = 0,60, 95 %-KI: 0,40–0,92, *p* = 0,02; OR = 0,99, 95 %-KI: 0,98–1,00, *p* = 0,04, respektiv), als prädiktiver Faktor für eine DLT ist die Sarkopenie anhand der vorliegenden Daten jedoch nicht geeignet.

Die Studien anderer Autoren zeigen, dass die anhand der Skelettmuskelquantität definierte Sarkopenie das Risiko einer schweren immunvermittelten Toxizität bei Patienten mit malignem Melanom erhöhen kann. Insbesondere bei Patienten mit Übergewicht und Sarkopenie sowie bei geringer Muskeldichte wurde ein solches Risiko beobachtet [6, 7]. In einer Metaanalyse konnte ein Zusammenhang zwischen der schweren immunvermittelten Toxizität unter Therapie mit ICI beim malignen Melanom und Sarkopenie allerdings nicht festgestellt werden [15].

Es ist bekannt, dass es Wechselwirkungen zwischen der Skelettmuskulatur und dem Immunsystem gibt [16, 17]. Die Skelettmuskulatur funktioniert wie ein endokrines Organ, indem sie eine spezifische Gruppe von Zytokinen und Peptiden synthetisiert und freisetzt, sog. Myokine. Auf diese Weise kann sie antiinflammatorische und immunprotektive Effekte erzielen [17, 18]. Es ist bekannt, dass das Myokin Interleukin-15 (IL-15) bei der Proliferation, Differenzierung und Reifung von natürlichen Killerzellen (NK) und T‑Zellen relevant ist [19, 20]. IL-15 könnte daher eine Rolle bei der Steigerung der antikörperabhängigen zellulären Zytotoxizität spielen [19]. Weitere Myokine wie Oncostatin M (OSM) und „secreted protein acidic and rich in cysteine“ (SPARC) scheinen ebenfalls über ein antitumorales Potenzial zu verfügen [21–23].

Die Skelettmuskulatur spielt neben ihrer Funktion als endokrines Organ auch eine Rolle bei der Metabolisierung von ICI [24]. Eine plausible Hypothese für eine erhöhte Toxizität bei Patienten mit Sarkopenie besteht darin, dass eine Abnahme der Skelettmuskelmasse die Pharmakokinetik von ICI beeinflussen könnte. Alle derzeit klinisch verwendeten therapeutischen Antikörper sind monoklonale Antikörper (mAbs) vom Typ Immunglobulin G (IgG). Da die Muskulatur zu den Hauptorganen für die Eliminierung von mAbs gehört, kann ein Verlust an Skelettmuskelmasse einen möglichen Grund für eine erhöhte Toxizität bei einer Therapie mit ICI darstellen [24]. Andere Autoren argumentieren, dass es durch den Verlust von Skelettmuskelmasse zu einer Abnahme des Verteilungsvolumens der monoklonalen Antikörper kommen könnte [25].

In dieser Studie hatte die Patientengruppe mit DLT im Vergleich zu der Patientengruppe ohne DLT niedrigere Werte für die Psoasmuskelparameter PMI und Gauge unter Therapie mit ICI. Die computertomographisch anhand der Psoasmuskulatur bestimmte Sarkopenie ist aber kein statistisch signifikanter Prädiktor für eine DLT. Zur Validierung der Ergebnisse dieser Studie bedarf es zukünftiger prospektiver Studien mit einer größeren Kohorte. Es sollten zudem weitere Methoden und jeweils einheitliche Grenzwerte zur Bestimmung der Sarkopenie in die Auswertung mit einbezogen werden. Limitiert wird die Studie zudem durch die Verwendung der Psoasmuskelparameter. Die standardisierte Messung entspricht allerdings aktuell der gesamten Muskelmasse auf der Schicht LWK3. Weitere Limitationen stellen die retrospektive Auswertung und die geringe Patientenzahl dar.

## Fazit für die Praxis

Im Rahmen einer personalisierten Medizin gewinnt auch die Körperzusammensetzung an Bedeutung. Einen Teilbereich der Körperzusammensetzung stellt hierbei die Muskulatur dar. Veränderungen der Skelettmuskulatur wirken sich auf die Ergebnisse onkologischer Therapien aus. In dieser Arbeit kann gezeigt werden, dass die Verminderung der Skelettmuskulatur mit einem vermehrten Auftreten von Toxizität unter Therapie von Melanompatienten mit ICI korrelieren könnte. Aufgrund dieser Daten sollte der Körperzusammensetzung in der Planung und Durchführung der dermatoonkologischen Therapie, insbesondere beim Einsatz von ICI, ein Augenmerk gelten.

## Abkürzungen

BMI: Body Mass index
BSA: Body surface area
CT: Computertomographie
CTCAE v5.0: Common Terminology Criteria for Adverse Events Version 5.0
DLT: Dosislimitierende Toxizität
irAE: Immunvermittelte Nebenwirkungen
ICI: Immuncheckpointinhibitor
IgG: Immunglobulin G
IL-15: Interleukin-15
LWK: Lendenwirbelkörper
mAb: Monoklonaler Antikörper
NK: Natürliche Killerzellen
OSM: Oncostatin M
PMA: Psoasmuskelfläche
PMD: Psoasmuskeldichte
PMI: Psoasmuskelindex
SPARC: Secreted protein acidic and rich in cysteine
